# Molecular epidemiology of *Mycoplasma pneumoniae* pneumonia in children, Wuhan, 2020–2022

**DOI:** 10.1186/s12866-024-03180-0

**Published:** 2024-01-17

**Authors:** Meng Xu, Ying Li, Yue Shi, Haizhou Liu, Xi Tong, Li Ma, Jie Gao, Qing Du, Hui Du, Di Liu, Xiaoxia Lu, Yi Yan

**Affiliations:** 1 CAS Key Laboratory of Special Pathogens and Biosafety, Center for Biosafety Mega-Science, Wuhan Institute of Virology, Chinese Academy of Sciences, Wuhan, 430071 China; 2 National Virus Resource Center, Wuhan Institute of Virology, Chinese Academy of Sciences, Wuhan, 430071 China; 3 Computational Virology Group, Center for Bacteria and Viruses Resources and Bioinformation, Wuhan Institute of Virology, Chinese Academy of Sciences, Wuhan, 430071 China Xiao Hong Shan No. 44, Wuchang District,; 4 Wuhan Institute of Virology, University of Chinese Academy of Sciences, Wuhan, 430071 China Xiao Hong Shan No. 44, Wuchang District,; 5 Wuhan Children’s Hospital, Tongji Medical College, Huazhong University of Science and Technology, Wuhan, 430014 China 100 Hongkong Road, Jiangan District, Hubei

**Keywords:** *Mycoplasma pneumoniae*, Metagenomics, Epidemiology, Genome diversity, Macrolide-Resistant

## Abstract

**Background:**

*Mycoplasma pneumoniae* (*M. pneumoniae*) is an important pathogen of community-acquired pneumonia in children. The factors contributing to the severity of illness caused by *M. pneumoniae* infection are still under investigation. We aimed to evaluate the sensitivity of common *M. pneumoniae* detection methods, as well as to analyze the clinical manifestations, genotypes, macrolide resistance, respiratory microenvironment, and their relationship with the severity of illness in children with *M. pneumoniae* pneumonia in Wuhan.

**Results:**

Among 1,259 clinical samples, 461 samples were positive for M. pneumoniae via quantitative polymerase chain reaction (qPCR). Furthermore, we found that while serological testing is not highly sensitive in detecting M. pneumoniae infection, but it may serve as an indicator for predicting severe cases. We successfully identified the adhesin P1 (P1) genotypes of 127 samples based on metagenomic and Sanger sequencing, with P1-type 1 (113/127, 88.98%) being the dominant genotype. No significant difference in pathogenicity was observed among different genotypes. The macrolide resistance rate of M. pneumoniae isolates was 96% (48/50) and all mutations were A2063G in domain V of 23S rRNA gene. There was no significant difference between the upper respiratory microbiome of patients with mild and severe symptoms.

**Conclusions:**

During the period of this study, the main circulating M. pneumoniae was P1-type 1, with a resistance rate of 96%. Key findings include the efficacy of qPCR in detecting M. pneumoniae, the potential of IgM titers exceeding 1:160 as indicators for illness severity, and the lack of a direct correlation between disease severity and genotypic characteristics or respiratory microenvironment. This study is the first to characterize the epidemic and genomic features of M. pneumoniae in Wuhan after the COVID-19 outbreak in 2020, which provides a scientific data basis for monitoring and infection prevention and control of M. pneumoniae in the post-pandemic era.

**Supplementary Information:**

The online version contains supplementary material available at 10.1186/s12866-024-03180-0.

## Background

*M. pneumoniae* belongs to the class of the Mollicutes and is one of the smallest known free-living cell organisms. *M. pneumoniae* infections affect both the upper and lower respiratory tracts and are endemic and epidemic worldwide in individuals of all ages [1]. In children, *M. pneumoniae* is a significant cause of community-acquired pneumonia (CAP), accounting for 10%-40% of cases [2]. The main symptoms of *M. pneumoniae* infection include headache, sore throat, fever, cough, and other common respiratory symptoms [3]. While *M. pneumoniae* infection can occur at any time of the year, its incidence is higher during late fall or winter [4]. The illness spreads via droplets and tends to spread within families or communities with an incubation period of 2 to 4 weeks. Incidences of *M. pneumoniae* infection showed three- to five- year epidemic cycles in multiple countries [5, 6], but the mechanism for such epidemic cycle remains unclear. A study argues that minor variation in the duration of immunity at the population level must be considered essential for the *M. pneumoniae* epidemic cycle [7].

The research on methods for detecting *M. pneumoniae* infection is important and necessary. Nucleic acid assays, such as qPCR, are prevalently employed in the detection of *M. pneumoniae* infections, owing to their high sensitivity and specificity [8, 9]. Serological assays are often preferred in clinical settings due to their rapid processing and ability to yield timely diagnostic results. However, it is important to note that, despite their widespread use in China, serological assays exhibit a relatively lower sensitivity compared to other diagnostic approaches [10, 11]. Therefore, in light of these limitations, it becomes imperative to reassess the value of serological testing in the accurate diagnosis of diseases.

*M. pneumoniae* isolates can be classified into two major genetic groups, designated subtype 1 (P1-type1) and subtype 2 (P1-type2), based on variations in repetitive elements RepMP2/3 and RepMP4 in the P1 protein gene [12]. The prevalence of genotypes varies in different countries and regions, and there are characteristic periodic shifts in genotypes [13, 14]. Multi-locus sequence typing (MLST) is another genotyping method for *M. pneumoniae*, which divides *M. pneumoniae* strains into 46 sequence types (STs) based on nucleotide polymorphisms of eight housekeeping genes (pubMLST, https://pubmlst.org/) [15]. The relationship between *M. pneumoniae* genotypes and the severity of clinical manifestations in infected patients is a widely debated issue.

For the treatment of *M. pneumoniae* infections in children, macrolides such as azithromycin and clarithromycin are generally considered as the first choice [16]. However, the widespread use of antibiotics has led to the emergence of macrolide-resistant *M. pneumoniae* (MRMP) [17]. This had led to an increasing number of severe *M. pneumoniae* pneumonia cases in children, which are more likely to develop serious pulmonary and extrapulmonary complications [18, 19]. Naturally occurring macrolide resistance in *M. pneumoniae* is due to mutations in various positions in the 23S rRNA, including C2617G, A2063G/C/T, and A2064G/C [19], with the transition mutation A2063G being the most commonly reported. Monitoring the prevalence and mutation types of MRMP will aid in clinical diagnosis and treatment.

In many areas of China, *M. pneumoniae* epidemic surveillance is not conducted, and there has been little data on *M. pneumoniae* genotypes, macrolide resistance, and epidemiology in Wuhan. Although the outbreak of *M. pneumoniae* pneumonia in Wuhan during the winter of 2019 was initially estimated, the monitoring of *M. pneumoniae* was delayed due to the impact of the coronavirus disease 2019 (COVID-19) outbreak. In recent years, there has been an increase in cases requiring hospitalization for treatment among children with *M. pneumoniae* infection. Therefore, the objective of this study was to elucidate the characteristics of this *M. pneumoniae* epidemic in Wuhan, including the incidence, genotype, drug resistance characterization of *M. pneumoniae*.

## Results

### Epidemiological characteristics of *M. pneumoniae* in Wuhan between 2020 and 2022

**Fig. 1:**
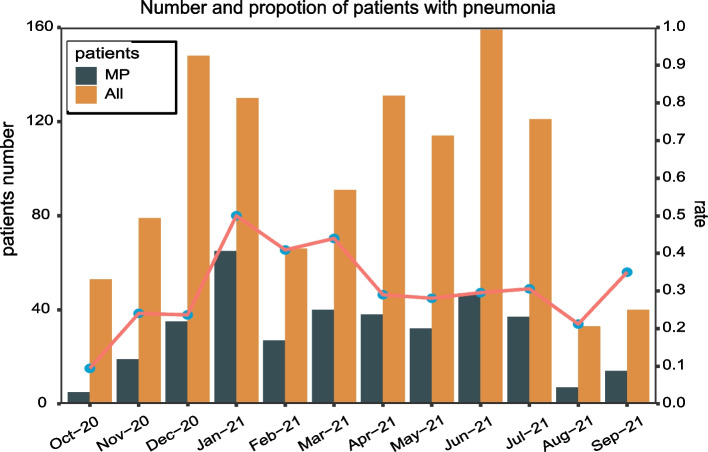
Epidemiological data about *M. pneumoniae* pneumonia. "MP" refers to patients diagnosed with *M. pneumoniae* pneumonia, while "All" refers to all patients with pneumonia

From October 2020 to March 2022, 1,259 clinical samples from children diagnosed with pneumonia in Wuhan were collected. Of the 1,259 children diagnosed with CAP, 461 (36.6%) cases were determined to be positive for *M. pneumoniae* via qPCR*.* After excluding patients with underlying diseases, 371 patients remained for further analysis. In terms of patient age, preschoolers (302/371, 81.4%) accounted for the majority of the *M. pneumoniae* pneumonia cases. However, the infection of *M. pneumoniae* in preschoolers, especially children aged 1–24 months, is milder in symptoms, while the infection in school-age children aged 6–11 years is more likely to develop into severe. There was no correlation between the gender of infected children and disease severity (Table 1). In terms of the clinical symptoms of patients, the most common symptoms observed in children included fever (260, 70.3%), paroxysmal cough (324, 87.3%), and in some cases wheezing (77, 20.8%). The median duration of fever was 4 days (Table 1). From the serological test results, it can be observed that patients with severe symptoms showed significantly lower qPCR cycle threshold (Ct) values and higher IgM titers. IgM titers ≥ 320 were considered to be a cause for concern, as patients with such titers are at a higher risk of developing severe disease (Table 1). In terms of the epidemic time of *M. pneumoniae* pneumonia, this study showed that *M. pneumoniae* infections have a high incidence during winter and early spring. *M. pneumoniae* infections can account for 40–50% of community-acquired pneumonia cases from January to March (Fig. 1).

**Table 1:** Characteristics of *M. pneumoniae* pneumonia collected from children with pneumonia in Wuhan, China, 2020–2022

	mild (*N* = 198)	Severe (*N* = 173)	*P*-value
Age			
1–24 M	67 (33.8%)	33 (19.1%)	< 0.001
2–5 Y	112 (56.6%)	90 (52.0%)	0.554
6-11Y	19 (9.6%)	39 (22.5%)	0.012
12-17Y	0 (0%)	11 (6.4%)	
Gender			
female	89 (44.9%)	78 (45.1%)	1
male	109 (55.1%)	95 (54.9%)	
Ct			
Mean (SD)	31.0 (4.27)	29.3 (4.37)	< 0.001
Missing	16 (8.1%)	51 (29.5%)	
Titer			
D40	68 (34.3%)	32 (18.5%)	< 0.001
D80	50 (25.3%)	17 (9.8%)	< 0.001
D160	31 (15.7%)	21 (12.1%)	0.212
D320	11 (5.6%)	31 (17.9%)	0.003
Missing	38 (19.2%)	72 (41.6%)	
P1 type			
Type 1	28 (14.1%)	85 (49.1%)	1
Type 2	3 (1.5%)	11 (6.4%)	

The association between disease severity and patient age, Ct values, and antibody titers was assessed using the t-test, while the relationship between disease severity and patient gender, as well as *M. pneumoniae* genotype, was analyzed using the two-sided Fisher's exact test

The most common extra-pulmonary manifestation was gastrointestinal (20.5%), others included vomit (10.8%), diarrhoea (11.9%) and liver function damage (3.2%). 6.2% of all children had some type of rash. Uncommonly, 14 (3.8%) children have experienced cardiovascular system complications, with three cases of Kawasaki disease and one case of pericardial effusion. Additionally, there were two cases of embolism and one case of encephalitis. Further analysis focusing on extra-pulmonary complications reveals that there are significant differences in gastrointestinal, dermatological, cardiovascular system complications between the mild and severe groups (Table 2).

**Table 2:** Comparison of extra-pulmonary symptoms in mild and severe *M*. pneumoniae pneumonia group

Diseases	Mild (*N* = 198)	Severe (*N* = 173)	*P*-value
**Gastrointestinal**	25(12.6%)	51(29.2%)	0.0001029
Vomiting	17(8.6%)	23(13.3%)	0.1967
Diarrhea	19(9.6%)	25(14.5%)	0.1999
Liver function damage	2(1.0%)	10(5.8%)	0.02112
**Dermatological**	6(3.0%)	17(9.8%)	0.01269
Rash	6(3.0%)	17(9.8%)	-
**Cardiovascular**	3(1.5%)	11(6.4%)	0.0146
Electrocardiographic Abnormality	3(1.5%)	7(4.0%)	0.1332
Kawasaki disease	0	3(1.7%)	-
Pericardial effusion	0	1(0.6%)	-
**Hematologic**	0	2(1.2%)	-
Thrombosis	0	2(1.2%)	-
**Neurological**	0	1(0.6%)	-
Encephalitis	0	1(0.6%)	-

Data are shown as numbers (%). Pearson's Chi-squared was used to compare extra-pulmonary symptoms in mild and severe *M. pneumoniae* pneumonia

### Evaluation of detection methods for *M. pneumoniae* infection

**Fig. 2:**
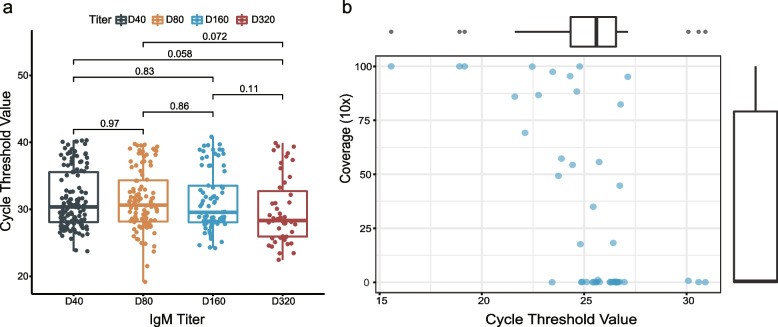
Relationships between antibody titers, Ct value and metagenome sequencing coverage of *M. pneumoniae* samples. **a**. Distribution of Ct values of samples with different antibody titers. Paired t-tests were used to measure the differences between groups. D40/D80/D160/D320 means *Mycoplasma pneumoniae* IgM antibody titer. **b**. Scatter plot of Ct values and 10 × genome coverage of metagenomic-sequenced samples

Serological testing and qPCR are common laboratory methods for detecting *M. pneumoniae* and a first-time IgM titer greater than 1:160 is considered as evidence of *M. pneumoniae* infection. The relationship between Ct values and IgM titers was analyzed, but no significant association was found (Fig. 2a). This finding suggests that IgM titers may not be a sensitive method for diagnosing *M. pneumoniae* infection, as they are inconsistent with nucleic acid detection results.

Metagenomic sequencing has been proven to be a powerful tool for various pathogens' identification. However, it is necessary to evaluate the efficiency of obtaining *M. pneumoniae* genomes through metagenomic sequencing. The reads were aligned to the reference genome of *M. pneumoniae* to determine the efficiency of metagenomic sequencing in obtaining the genome. While genome coverage appeared to be higher in patients with severe symptoms, this difference was statistically significant at sequencing depth greater than 30 × (Figure S1 a). Despite the amount of sequenced data is enormous, only a small portion of reads come from *M. pneumoniae* (Figure S1 b). Meanwhile, except for some samples with extremely low Ct values, the Ct value seemed to have little relationship with the coverage of the *M. pneumoniae* genome (Fig. 2b). This suggests that performing metagenomic sequencing directly on clinical samples may not be the optimal approach for obtaining the *M. pneumoniae* genome.

### The genomes of *M. pneumoniae* are highly conserved

**Fig. 3:**
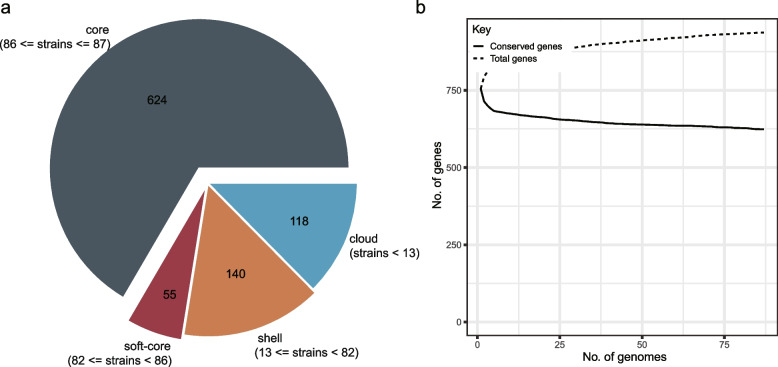
Pan-genomes of *M. pneumoniae*. **a**. The pan-genome pie chart shows the breakdown of genes and the number of isolates they are present in. **b**. Heaps law chart representation regarding conserved genes vs total genes in 87 genomes

To obtain sufficient variation for phylogenetic analysis, we performed pan-genome analysis on 87 genomes of *M. pneumoniae*. The results showed that the total pan-genome was composed of 937 homologous genes (homologs) with a core genome of 624 (66.6%) conserved homologs presenting in 100% of all *M. pneumoniae* isolates (Fig. 3a). Examination of the gene accumulation curve (Fig. 3b) revealed that as the number of genomes increased, the number of core genes quickly decreased to a stable level, indicating a highly conserved genome in *M. pneumoniae*. The total number of genes also approached saturation, indicating that the pan-genome is open but still relatively constrained.

### Prevalence of *M. pneumoniae* genotypes in Wuhan

Since there were few studies on *M. pneumoniae* genotype surveillance in Wuhan previously, it is crucial to understand the epidemic trend of *M. pneumoniae* genotypes and the formulation of prevention and control measures accordingly.

**Fig. 4:**
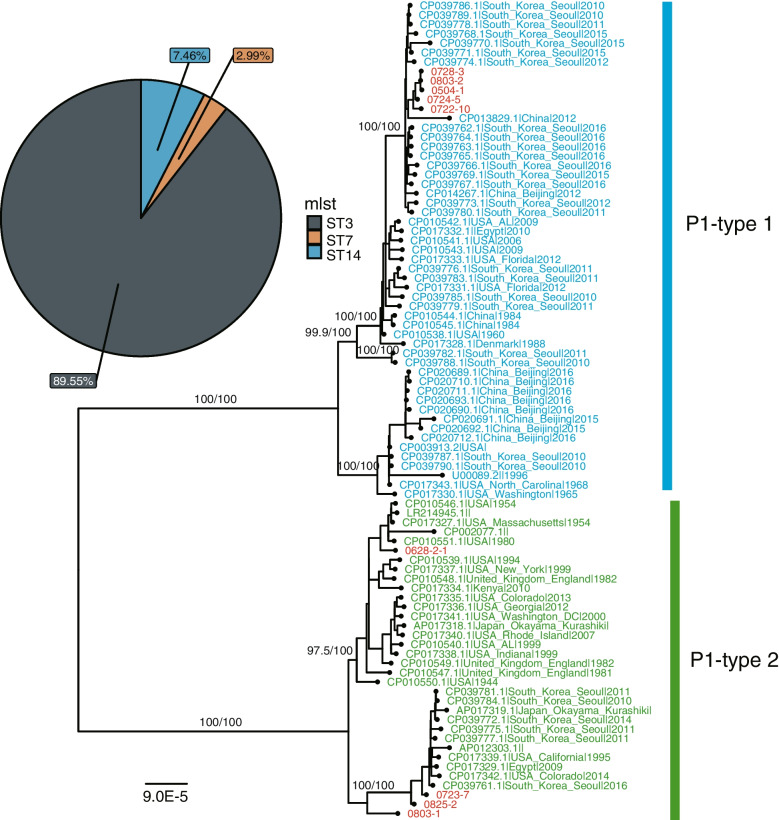
Prevalence of *M. pneumoniae* genotypes of the present epidemic. Maximum likelihood phylogenetic tree of the core genome of 87 M*. pneumoniae* isolates, of which 80 are from GenBank. The blue branches represent P1-type1 of *M. pneumoniae*, and the green branches represent P1-type 2. Marked in red are samples or sequence types in this study. The pie chart represents the proportion of *M. pneumoniae* MLST genotypes. ST, sequence typing

The results of the phylogenetic analysis showed that the *M. pneumoniae* epidemic was a mixed infection of two P1 genotypes (Fig. 4). Among the 127 P1-type strains identified, 113 (88.98%) were P1-type 1 and 14 were P1-type 2 (11.02%). Additionally, the MLST genotype of *M. pneumoniae* was successfully distinguished in 67 samples by mNGS and Sanger sequencing, of which 2 (3%) were ST-7, 5 (7.5%) were ST-14, and 60 (89.5%) were ST-3 (Fig. 4).

To explore the differences in the pathogenic potential of different genotypes, we counted the number of mild and severe patients with different P1 genotypes. In sample P1-type 1, there are 28 samples from mild patients and 85 samples from severe patients. In sample P1-type 2, there are 3 samples from mild patients and 11 samples from severe patients. The result of Fisher's exact test showed that *M. pneumoniae* genotype had no effect on disease severity (*p*-value = 1) (Table 1).

### Comparison of microbial composition and diversity in different clinical samples

**Fig. 5:**
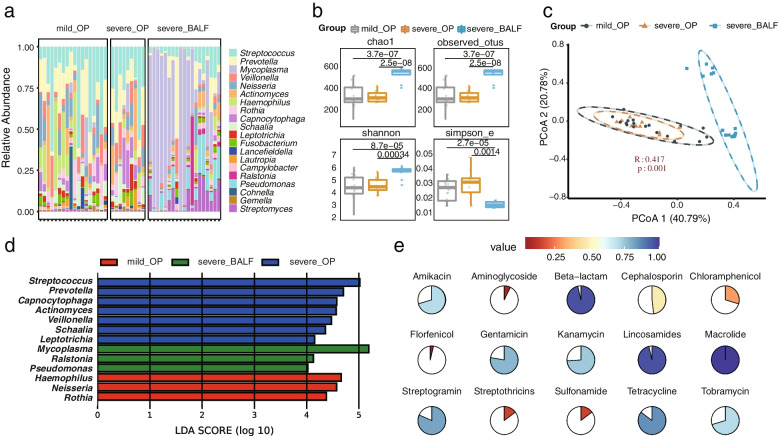
Comparison of microbial composition and diversity in different clinical samples. **a** The microbial composition of 46 individual donors is shown, with only the top 20 microbial genera with the highest abundance displayed. **b** The alpha diversity of OP microbiota from patients with mild and severe symptoms and BALF in critically ill patients was calculated according to the genus-level Chao1 index, observed OTUs, Shannon index, and Simpson index. Paired t-tests were used to compare the differences between groups. **c** Beta diversity of OP microbiota from patients with mild and severe symptoms and BALF in critically ill patients. Differences between groups were tested by PERMANOVA. **d** LEfSe identifies microbial markers in the three clinical subgroups with a linear LDA score greater than 4 considered a biomarker. **e** The pie chart displays the number of oropharyngeal swab samples with detected resistance genes relative to the total number of samples

As we did not observe a correlation between *M. pneumoniae* genotypes and the severity of illness, we wondered to investigate whether there are differences in the respiratory microbiota of patients with different disease severities. Our results showed that although infected with *M. pneumoniae*, the top 20 most abundant genera in OP samples were mainly colonized conditional pathogens, such as *Streptococcus, Prevotella, Veillonella and Neisseria* (Fig. 5a). Alpha diversity analysis did not show any significant difference in the number of microbial genera (Chao1 and observed OTU index) and the within-sample diversity (Shannon and Simpson index) among OP samples (Fig. 5b). Beta diversity analysis also revealed no differences in microbial composition among OP samples (Fig. 5C). However, there were differences in microbial diversity and composition between BALF and OP samples, which were attributed to sampling site (Fig. 5b, c). The relative abundance of genera did not differ significantly between OP samples from mild and severe patients (Figure S2). Linear discriminant analysis (LDA) scores showed *Mycoplasma* as a marker genus for BALF (Fig. 5d), while biomarkers in OP included genera such as like *Streptococcus*, *Prevotella*, *Neisseria*, *Haemophilus*, among others. Therefore, it was concluded that the upper respiratory microbiome of *M. pneumoniae* infected patients was not affected by the severity of symptoms.

### Antibiotic resistance mutations in 23S rRNA gene of *M. pneumoniae* and resistance genes in the microbiome

Macrolide-resistant *M. pneumoniae* is a potential factor leading to intractable *M. pneumoniae* pneumonia, and the resistance rate of *M. pneumoniae* in China is very high. Therefore, monitoring the resistance rate of *M. pneumoniae* is particularly important. We successfully amplified 23S rRNA sequences from 50 clinical samples, 48 (96%) of which contained the A2063G mutation. Besides, drug resistance mutations at positions 2064 and 2617 were not found in this study.

De novo assembly of other microorganisms from the OP samples also revealed the presence of resistance genes to various antibiotics, including Tetracycline, Macrolide, Amikacin, Gentamicin, Kanamycin, Tobramycin, Lincosamides, Streptogramin, Cephalosporin, and Beta-lactam in many OP samples. However, these antibiotic resistance genes were rarely identified in BALF samples (Fig. 5e, Table S3). The sample shows a remarkably high frequency of resistance genes to three types of antibiotics: Tetracycline, Macrolide, and Beta-lactam. The presence of these resistance genes in other microorganisms may pose challenges for the treatment of *M. pneumoniae* co-infection. Notably, these resistance genes were frequently detected in genera such as *Streptococcus*, *Enterococcus*, *Pseudomonas*, *Escherichia* and *Prevotella*, which were less abundant in BALF and thus rarely detected (Table S4).

## Discussion

In mainland China, most studies related to *M. pneumoniae* have been conducted in Beijing and North China [20, 21], while few have been conducted in other regions. Xue et al. were the first to report the genotype and antimicrobial resistance of *M. pneumoniae* in 6 regions simultaneously [22]. In this study, we assessed the genotype and macrolide resistance mutation of *M. pneumoniae* in children in Wuhan for the first time. More importantly, this is the first surveillance data on *M. pneumoniae* in Wuhan since the COVID-19 outbreak. *M. pneumoniae* is underestimated because of its self-limiting nature, but it still causes up to 40% of CAP and contributes to the social burden. Our results showed that *M. pneumoniae* pneumonia accounted for 36.6% of community-acquired pneumonia cases, making it a respiratory pathogen that deserves our close attention. Our study inferred that 88.98% of infected *M. pneumoniae* strains were classified as P1-type 1 and 11.02% were P1-type 2. This result was similar to a previous study in that 89.1% of *M. pneumoniae* in Beijing were classified as P1-type 1 in 2018 [22]. This study is the first to investigate *M. pneumoniae* genotypes in Wuhan, which provides a foundation for future studies on *M. pneumoniae* genotype shift cycles in Wuhan. What’s more, it remains controversial whether clinical manifestations of lower respiratory tract infections (LRTI) in children differ between the two *M. pneumoniae* P1 genotypes. Some studies indicated that the two P1 genotypes may have different pathogenic potentials and that LRTI with P1-type 2 strains may have a more severe disease course than those with P1-type 1 strains in children [23, 24]. However, the result in other areas may be quite the opposite [25]. In the present study, we did not find any specific genotype associated with more severe clinical symptoms.

Given that many regions lack systematic monitoring of *M. pneumoniae* pneumonia, we recommend establishing genotype and macrolide resistance monitoring systems for *M. pneumoniae* in more cities in China to facilitate the study of genotype switching and macrolide resistance and their relationship with clinical manifestations. Furthermore, the development of a novel method to directly obtain the P1 genotype of *M. pneumoniae* from clinical samples without sequencing is necessary, which would facilitate the monitoring of *M. pneumoniae*.

The serological assay is the most commonly used method for detecting *M. pneumoniae* infection. A fourfold increase in IgG titer in acute and convalescent sera is considered a diagnostic criterion of acute *M. pneumoniae* respiratory infection, while an initial IgM antibody detection titer greater than or equal to 1:160 serve as a criterion for recent *M. pneumoniae* infection [26]. If IgM is measured at least 7 days after the onset of symptoms, acute infection can be detected. However, if the test is performed earlier, the result may be negative [27]. It is risky to diagnose acute *M. pneumoniae* respiratory infection based solely on a single IgM test, and it is also difficult to implement in the practical diagnosis of children. qPCR is suitable for detecting *M. pneumoniae* infection, while IgM titers greater than 1:160 can be used as a predictor of severity. The traditional way of obtaining *M. pneumoniae* genome is through culture and whole genome sequencing. However, this approach is time-consuming and has a low yield. Therefore, we attempted to perform metagenomic sequencing directly on clinical specimens. Nevertheless, this method only provided a limited amount of genomic information. Out of the large amount of data produced by sequencing, only a few reads were related to *M. pneumoniae*. Therefore, this strategy is highly dependent on samples with a high *M. pneumoniae* abundance to obtain high-quality genomes.

Since the first identification of an MRMP strain in pediatric patients in Japan in 2000 [17], there has been a notable increase in the prevalence of such strains, highlighting a growing concern for public health and antibiotic resistance management. The emergence of MRMP brought challenges in the clinical treatment of *M. pneumoniae* infection [28]. The highest proportion of MRMP infections was observed in the Western Pacific region worldwide, with an increasing trend observed over the years. China had the highest proportion of MRMP infections within this region, followed by Japan and other countries [29]. Mutation A2063G is the most common mutation in macrolide-resistant *M. pneumoniae*, with a high prevalence in previous reports [28, 30]. Specific mutations at the V domain of 23S rRNA of *M. pneumoniae* may determine macrolide resistance phenotypes. For instance, mutations at positions 2063 and 2064 are associated with high-level resistance, while mutations at positions 2067 and 2617 are related to low-level macrolide resistance [31–33]. In this study, all the mutations detected in *M. pneumoniae* were of the A2063G and the proportion of this mutation in these strains was as high as 96%. In addition, multiple antibiotic-resistance genes were detected in colonized microorganisms in the respiratory tract, indicating that the widespread use of antibiotics has resulted in more and more microbial resistance, which may pose increasing challenges for treating respiratory infectious diseases.

It must be acknowledged that this study has certain limitations. Firstly, no specific genotype of *M. pneumoniae* was found to be associated with more severe clinical symptoms in our analysis, but the small sample size may have constrained our findings. Further research, incorporating larger datasets from various regions, is essential to validate these observations. A notable limitation of this study is the insufficient identification of macrolide-sensitive *M. pneumoniae* strains, which precludes the analysis of any correlation between macrolide resistance and disease severity. Furthermore, the identification of only one type of resistance mutation limits the ability to compare the pathogenic differences among various resistance mutations.

## Conclusion

In conclusion, this study laid a foundation for the cyclic study of *M. pneumoniae* genotype conversion in Wuhan, and the main circulating *M. pneumoniae* from 2020 to 2022 is P1-type 1. This study also underscored the efficacy of qPCR as a sensitive method for detecting *M. pneumoniae* infections. The finding that an IgM titer exceeding 1:160 may serve as a predictive angle for assessing the severity of the illness, adding a valuable perspective to clinical assessment. Significantly, our results revealed no direct correlation between the severity of *M. pneumoniae* pneumonia and its genotypic characteristics or the respiratory microenvironment, suggesting that other factors may influence disease progression. The high prevalence of macrolide resistance mutations identified presented a growing challenge for treatment, emphasizing the need for continued surveillance and novel therapeutic approaches. These findings contribute to a deeper understanding of *M. pneumoniae* infections and set the stage for future research into effective prevention and control strategies.

## Materials and methods

### Patients and specimens

A total of 1259 children admitted to Wuhan Children's Hospital with pneumonia between October 2020 and March 2022 were enrolled in this study. Among them, 511 were clinically diagnosed with *M. pneumoniae* infection based on the colloidal gold assay for *M. pneumoniae* IgM titer of ≥ 1:160 [34]. After obtaining written informed consent from patients or their parents, 417 oropharyngeal swabs (OP) and 94 bronchoalveolar lavage fluid samples (BALF) were collected. OP samples were collected from each patient within 24 h after admission. Electronic bronchoscopy was performed when necessary, and BALF samples were collected during these procedures simultaneously within the hospitalization period. The samples were sent to Wuhan Institute of Virology, Chinese Academy of Sciences for analysis. Each OP sample was stored in 3 mL of viral transport media at -80℃ and BALF was directly stored at -80℃ before use. Children with immunodeficiency, chronic lung disease, neurological disorders and gastrointestinal and urinary tract infections were excluded.

### Real-time PCR, MLST and 23S rRNA PCR

Nucleic acid was extracted from OP and BALF samples (200 μL) using a BeaverBeads™ Viral DNA/RNA Kit (Suzhou, China) according to the manufacturer’s instructions. qPCR was utilized specifically for the detection of *M. pneumoniae*, while PCR was performed to amplify the target genes using DNA from OP and BALF samples. The primers used for qPCR [35] and PCR, along with their corresponding target genes, are detailed in Supplementary Table S1. MLST was conducted based on the scheme described in the PubMLST website (https://pubmlst.org/mpneumoniae/) developed by Brown et al. [15]. The primers used for amplifying the 23S rRNA fragment were designed in-house. Each reaction of qPCR was conducted in a final volume of 10 μL containing 2 × SYBR® Green Realtime PCR Master Mix (5 μL), specific primers (10 μM × 0.4 μL), 1–2 μL of template DNA, and ddH2O up to 10 μL. The amplification conditions were as follows: 95℃ for 30 s; 40 cycles of 95℃ for 5 s, 55℃ for 10 s and 72℃ for 15 s. Each reaction of PCR was conducted in a final volume of 50 μL containing 2 × TsingKe® Master Mix (Beijing, China), specific primers (10 μM × 1 μL), 1–2 μL of template DNA, and ddH2O up to 50 μL. The amplification conditions were as follows: 95℃ for 3 min; 30 cycles of 94℃ for 30 s, 55℃C for 30 s and 72℃ for 30–80 s; with a final extension step of 72℃ for 5 min. All amplification products were purified using TIANgel Midi Purification Kit (DP209) (Beijing, China) and sequenced in Tsingke Biotech (Beijing, China).

### Metagenome sequencing, classifications and microbiome analysis

DNA library was constructed using VAHTS Universal Plus DNA Library Prep Kit for Illumina® ND617 (Nanjing, China). Library circularization and next-generation sequencing (NGS) of all samples were performed using the MGI T7 platform. 46 samples with Ct value below 27.5 were selected for metagenome next-generation sequencing (mNGS) and paired-end 150-bp reads were used. Reads classification from metagenomic sequencing was conducted using Kraken2 v2.1.2 [36] software with the kraken2 nt database, applying a confidence threshold of 0.05. The report files of taxonomic classifications were visualized by the Pavian software tool [37]. Alpha diversity analysis was performed using QIIME [38], and the beta diversity analysis was performed in R using the vegan package [39]. Inter-group microbial differential analysis was performed using LEfSe v1.1.2 [40], and an linear discriminant analysis (LDA) score greater than 4 was considered to indicate significant differences.

### Pan-genome and phylogenetic analysis

Paired-end reads were mapped to either the M129 or FH reference genome (NCBI accession numbers: NC_000912.1 and CP010546.1, respectively) using Snippy v4.6.0 (https://github.com/tseemann/snippy) with default parameters. When mapping reads to a reference genome, Snippy could generate a consensus sequence of the reference genome. We annotated the consensus sequences with high sequencing depth (> 10) and coverage (> 95%) using Prokka v1.14.5 [41]. A pan-genome was constructed using draft assemblies of the 87 M*. pneumoniae* isolates, which included 78 genomes from GenBank and 9 consensus sequences generated in this study. Genomic feature files output from Prokka in gff3 format for each strain were used for the pan-genome analysis using Roary v3.13.0 [42]. Roary was run with the "-e" flag to generate a multi-FASTA alignment of core genes using PRANK and a minimum blastp identity value of 95%. Summary of the pan-genome composition provided by Roary was visualized in Fig. 3A using the open source Python script 'roary_plots.py' (available at https://github.com/sanger-pathogens/Roary/tree/master/contrib/roary_plots). Gene diversity estimation and visualization presented in Fig. 3B was performed using the native Rscript from Roary (create_pan_genome_plots.R). Alignment of core genes was used to build a maximum likelihood (ML) phylogenetic tree by IQ-tree2 v2.2.0_beta with ModelFinder (model K3Pu + F + I + G4) [43, 44]. The ML tree was visualized by FigTree v1.4.4 (http://tree.bio.ed.ac.uk/software/figtree/).

### De novo assembly and detection of resistant genes

mNGS reads were de novo assembled using Megahit v1.2.9 [45]. Mass screening for antimicrobial resistance was performed on the contigs assembled by Megahit using AMRFinderPlus v3.10.30 [46]. Following this, the frequencies of these antimicrobial resistance genes across all samples were calculated.

### Statistical analysis

The data were expressed as the mean and standard deviation or as a rate (%). Group comparisons were conducted using t-test, PERMANOVA or linear discriminant analysis (LDA). For categorical variables, two-sided proportions test or Fischer's exact test was used. A p-value of less than 0.05 was considered statistically significant. LDA was performed by LEfSe and other statistical analyses were performed by using R v4.1.3 [47].

### Supplementary Information

**Additional file 1.**

**Additional file 2.**

**Additional file 3.**

**Additional file 4.**

**Additional file 5.**

**Additional file 6.**

## Data Availability

The bioproject number for the raw sequencing data reported in this paper is PRJCA012901 (available at http://bigd.big.ac.cn).
