# Supplementary material for: Molecular epidemiology of Mycoplasma pneumoniae pneumonia in children, Wuhan, 2020–2022
Source: BMC Microbiol. 2024 Jan 17;24:23. doi: 10.1186/s12866-024-03180-0 (PMC10792977; doi:10.1186/s12866-024-03180-0)
Supplement: Supplementary file 1 — Additional file 1. [file 12866_2024_3180_MOESM1_ESM.pdf]

Figure S1.

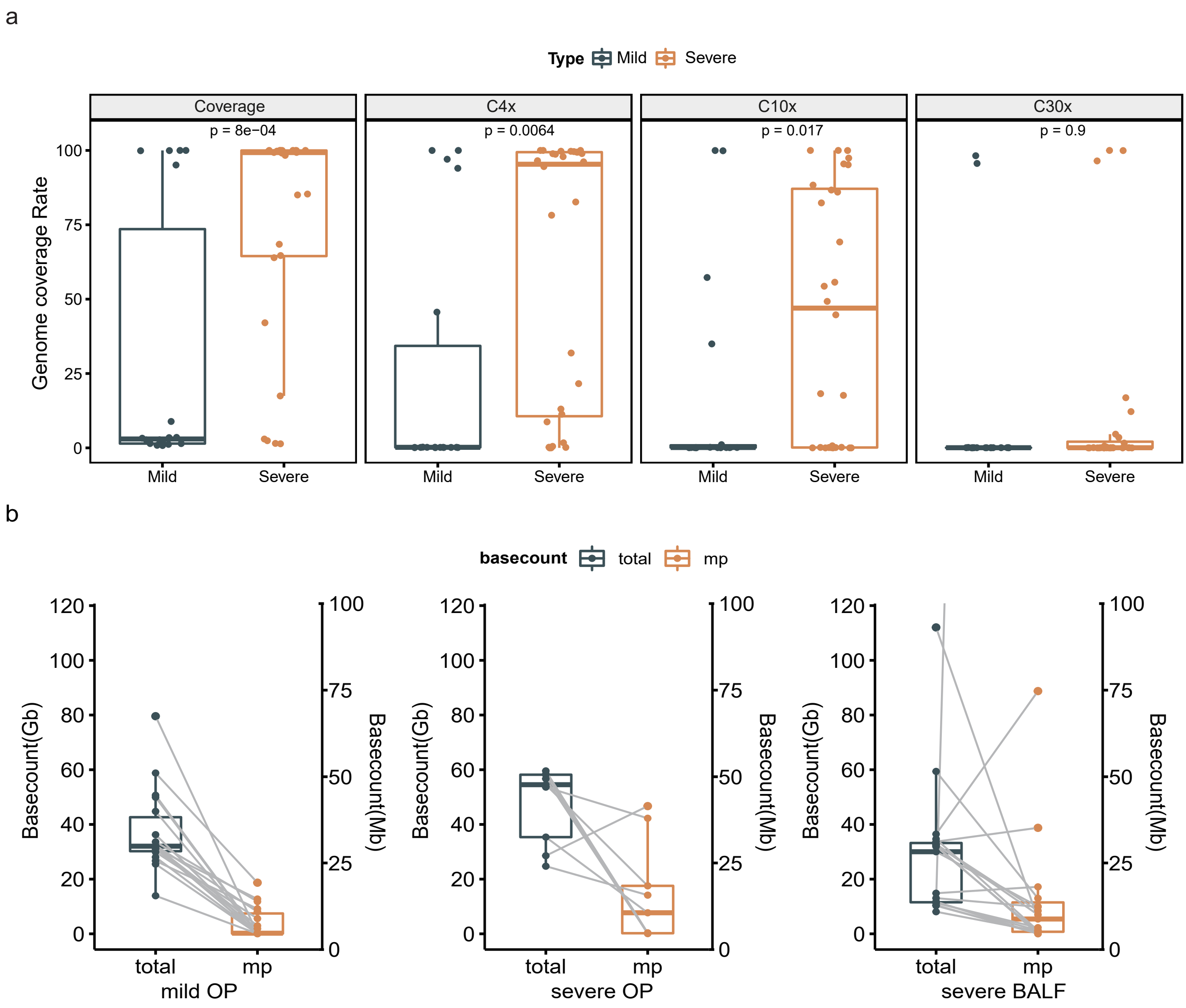

Figure S1. Evaluation of the *M. pneumoniae* genome obtained by metagenomic sequencing. a. Genome coverage of *M. pneumoniae* achievable with samples from patients with mild and severe symptoms. b. The relationship between the number of *M. pneumoniae* bases and the total number of bases in whole metagenomic sequencing.
