# Supplementary material for: Molecular epidemiology of Mycoplasma pneumoniae pneumonia in children, Wuhan, 2020–2022
Source: BMC Microbiol. 2024 Jan 17;24:23. doi: 10.1186/s12866-024-03180-0 (PMC10792977; doi:10.1186/s12866-024-03180-0)
Supplement: Supplementary file 2 — Additional file 2. [file 12866_2024_3180_MOESM2_ESM.pdf]

Figure S2.

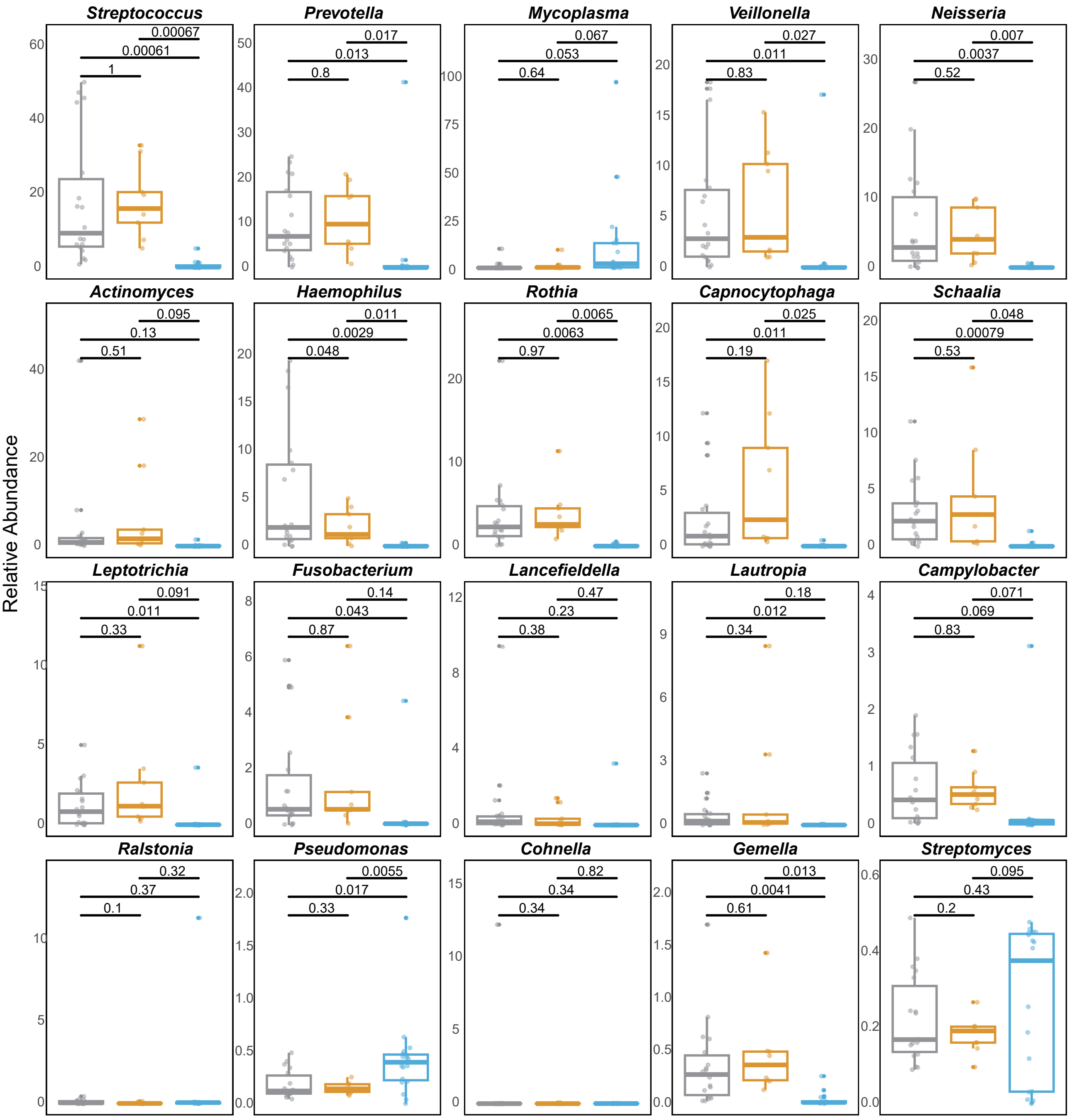

Figure S2. Comparison of the relative abundance of top20 microbial genera in different clinical groups. Microorganisms with significant differences in abundance among the 3 clinical subgroups were detected by the paired t-test.
