# Supplementary material for: Molecular epidemiology of Mycoplasma pneumoniae pneumonia in children, Wuhan, 2020–2022
Source: BMC Microbiol. 2024 Jan 17;24:23. doi: 10.1186/s12866-024-03180-0 (PMC10792977; doi:10.1186/s12866-024-03180-0)
Supplement: Supplementary file 3 — Additional file 3. [file 12866_2024_3180_MOESM3_ESM.pdf]

Table S1. Primers for qPCR and PCR.

gene	geneid	F	R	amplicon(bp)
gene-1	ppa	CGCTGACCAAGCCTTTCTAC	CACTCCAAACTTTGCACTCCC	256
gene-2	pgm-1	AGCACCTTGCACGATGAAGA	GGGTAGATCCACTTCCACTCC	551
gene-3	pgm-2	GCTACGTATGACTTAGCACCG	GGGCCATGCCATCAGATACA	450
gene-4	gyrB	TTGTCCCGGACTTTACCGTG	TGTTTTCGACAGCAAAGCGG	429
gene-5	gmk	GGTCGATACAGGGAGAATTTTTGTC	CAATTTGTTGACATTCCTTCTCAGC	432
gene-6	glyA-1	GGAATTGCAAAGACAAAGAGATTGC	GGTTTTACCTCCCGAGCAAC	356
gene-7	glyA-2	GAGCGCTTTAGTGCAATTGC	TGACAACCCGGAAAGACACC	187
gene-8	atpA	GAAAACAACGTGCAGGGGAT	GGTAAAGCGGTAATCGAGCC	745
gene-9	arcC	CCCCATCAAGCCGTGTACTT	TTGGGCAATAATGGCCGTCT	570
gene-10	adk	GTAGCCAACACCACCGGATT	ACGGTGTCTTCGTAAAGCGT	473
gene-11	23S	GGGTAAATTCCGTCCCGCTT	CGGTCCTCTCGTACTAGAAGC	681
gene-12	P1	TTACCTTAGGACTTGCCATTGGA	CGCAAACCCAGCCTTCAA	68
