# Supplementary material for: Molecular epidemiology of Mycoplasma pneumoniae pneumonia in children, Wuhan, 2020–2022
Source: BMC Microbiol. 2024 Jan 17;24:23. doi: 10.1186/s12866-024-03180-0 (PMC10792977; doi:10.1186/s12866-024-03180-0)
Supplement: Supplementary file 4 — Additional file 4. [file 12866_2024_3180_MOESM4_ESM.pdf]

Table S2. Sequencing data details for each sample.

| Patients ID | Gender | Age | Collection Time | Ct Value | Disease Severity | basecount/G | mapping rate | mapping reads | Coverage | 4x | 10x | 30x | mlst | Classified | Chordate | Artificial | Unclassified | Microbial | Bacterial | Viral | Fungal | Protozoan |
|---|---|---|---|---|---|---|---|---|---|---|---|---|---|---|---|---|---|---|---|---|---|---|
| patients1 | Female | 6Years | 2021/2/2 | 26.73 | Mild | 31.68 | 0 | 893 | 2.74 | 0.16 | 0.09 | 0.06 | | 97.50% | 70.20% | 0.00% | 2.49% | 13.50% | 9.43% | 1.73% | 0.06% | 0.01% |
| patients2 | Female | 4Years and 3Months | 2021/2/2 | 26.64 | Mild | 32.10 | 0 | 677 | 0.79 | 0.19 | 0.11 | 0.06 | | 96.40% | 68.30% | 0.00% | 3.62% | 15.90% | 12.70% | 0.83% | 0.07% | 0.01% |
| patients3 | Male | 2Years | 2021/2/4 | 26.5 | Mild | 50.68 | 0 | 980 | 3.53 | 0.21 | 0.13 | 0.05 | | 98.70% | 72.20% | 0.00% | 1.27% | 12.20% | 9.10% | 0.73% | 0.05% | 0.01% |
| patients4 | Male | 1Years and 1Months | 2021/2/4 | 25.46 | Mild | 49.93 | 0 | 532 | 2.71 | 0.14 | 0.1 | 0.05 | | 99.40% | 77.70% | 0.00% | 0.64% | 6.50% | 3.82% | 0.38% | 0.05% | 0.01% |
| patients5 | Male | 1Years and 2Months | 2021/3/2 | 25.59 | Mild | 79.58 | 0 | 983 | 8.88 | 0.16 | 0.06 | 0.04 | | 99.80% | 80.20% | 0.00% | 0.19% | 3.16% | 0.93% | 0.10% | 0.05% | 0.01% |
| patients6 | Male | 2Years and 2Months | 2021/3/23 | 26.96 | Mild | 36.25 | 0 | 615 | 1.37 | 0.19 | 0.12 | 0.07 | | 96.80% | 70.30% | 0.00% | 3.16% | 13.80% | 10.60% | 0.73% | 0.07% | 0.02% |
| patients7 | Female | 1Years and 4Months | 2021/5/8 | 26.38 | Mild | 13.92 | 0 | 198 | 0.86 | 0.08 | 0.05 | 0.03 | | 99.50% | 81.50% | 0.00% | 0.50% | 4.17% | 1.90% | 0.12% | 0.06% | 0.01% |
| patients8 | Female | 2Years and 3Months | 2021/5/24 | 25.6 | Mild | 28.04 | 0 | 618 | 3.45 | 0.12 | 0.06 | 0.04 | | 99.10% | 77.00% | 0.00% | 0.87% | 4.55% | 2.25% | 0.13% | 0.04% | 0.01% |
| patients9 | Male | 4Years and 1Months | 2021/6/28 | 23.89 | Mild | 26.71 | 0.03 | 61018 | 99.95 | 97.04 | 57.28 | 0.04 | 3 | 99.70% | 78.70% | 0.00% | 0.26% | 2.94% | 0.70% | 0.05% | 0.05% | 0.01% |
| patients10 | Male | 1Years and 9Months | 2021/7/5 | 24.86 | Mild | 25.49 | 0 | 391 | 3.25 | 0.08 | 0.03 | 0.02 | | 99.80% | 77.40% | 0.00% | 0.25% | 4.45% | 0.78% | 0.03% | 0.04% | 0.01% |
| patients11 | Female | 10Months and 11Days | 2021/7/13 | 25.67 | Mild | 31.99 | 0.01 | 19649 | 95.10 | 45.64 | 1.08 | 0.05 | | 99.20% | 78.50% | 0.00% | 0.80% | 6.37% | 4.21% | 0.08% | 0.05% | 0.01% |
| patients12 | Female | 5Years and 9Months | 2021/7/14 | 25.44 | Mild | 32.70 | 0.02 | 46571 | 99.90 | 94 | 34.95 | 0.07 | | 95.60% | 68.40% | 0.00% | 4.42% | 14.70% | 10.60% | 0.91% | 0.07% | 0.01% |
| patients13 | Male | 10Months and 1Days | 2021/7/24 | 19.16 | Mild | 28.56 | 0.15 | 311328 | 100.00 | 99.99 | 99.97 | 98.22 | 3 | 99.70% | 77.50% | 0.00% | 0.33% | 4.03% | 0.72% | 0.05% | 0.05% | 0.01% |
| patients14 | Male | 2Years and 10Months | 2021/7/25 | 26.43 | Mild | 59.58 | 0 | 2426 | 1.18 | 0.26 | 0.2 | 0.14 | | 96.90% | 56.80% | 0.00% | 3.15% | 27.50% | 23.70% | 1.63% | 0.06% | 0.01% |
| patients15 | Male | 1Years and 10Months | 2021/7/28 | 22.46 | Mild | 53.64 | 0.08 | 281717 | 100.00 | 99.99 | 99.89 | 95.63 | 3 | 99.10% | 78.00% | 0.00% | 0.86% | 5.57% | 2.87% | 0.27% | 0.05% | 0.01% |
| patients16 | Female | 4Years and 7Months | 2021/7/30 | 25.75 | Mild | 56.71 | 0 | 871 | 1.46 | 0.18 | 0.13 | 0.07 | | 98.40% | 74.40% | 0.00% | 1.61% | 8.59% | 5.94% | 0.49% | 0.05% | 0.01% |
| patients17 | Female | 6Months and 17Days | 2021/8/4 | 26.27 | Mild | 10.29 | 0 | 943 | 1.44 | 0.16 | 0.14 | 0.08 | | 93.40% | 39.30% | 0.00% | 6.59% | 46.20% | 39.50% | 4.87% | 0.08% | 0.01% |
| patients18 | Male | 2Years and 11Months | 2021/8/4 | 26.62 | Mild | 10.47 | 0 | 528 | 1.96 | 0.17 | 0.1 | 0.05 | | 95.30% | 62.40% | 0.00% | 4.69% | 19.20% | 14.20% | 2.08% | 0.08% | 0.02% |
| patients19 | Male | 8Years and 6Months | 2021/3/1 | 26.54 | Severe | 44.77 | 0 | 2044 | 17.47 | 0.51 | 0.12 | 0.05 | | 98.70% | 74.50% | 0.00% | 1.27% | 8.84% | 6.03% | 0.38% | 0.05% | 0.01% |
| patients20 | Female | 9Years and 3Months | 2021/6/15 | 21.61 | Severe | 31.24 | 0.04 | 84870 | 99.98 | 99.71 | 85.99 | 0.6 | 3 | 99.80% | 80.70% | 0.00% | 0.24% | 3.55% | 0.22% | 0.01% | 0.05% | 0.01% |
| patients21 | Male | 4Years and 4Months | 2021/6/24 | 26.42 | Severe | 29.92 | 0.02 | 37152 | 99.09 | 82.67 | 18.23 | 0 | | 99.80% | 80.00% | 0.00% | 0.15% | 2.71% | 0.18% | 0.02% | 0.05% | 0.01% |
| patients22 | Male | 8Years and 5Months | 2021/6/28 | 23.74 | Severe | 32.35 | 0.02 | 53810 | 99.33 | 96.54 | 49.25 | 0.08 | 7 | 93.90% | 60.70% | 0.00% | 6.13% | 22.10% | 17.60% | 1.72% | 0.08% | 0.02% |
| patients23 | Male | 1Years and 7Months | 2021/7/5 | 23.46 | Severe | 58.79 | 0.03 | 124670 | 99.71 | 99.61 | 97.43 | 16.87 | 3 | 98.40% | 72.50% | 0.00% | 1.61% | 11.80% | 9.18% | 0.25% | 0.05% | 0.01% |
| patients24 | Male | 7Years and 2Months | 2021/7/13 | 24.9 | Severe | 31.09 | 0.01 | 12685 | 85.34 | 21.6 | 0.19 | 0.05 | | 98.40% | 76.30% | 0.00% | 1.61% | 7.90% | 5.18% | 0.25% | 0.06% | 0.01% |
| patients25 | Female | 5Years and 6Months | 2021/7/14 | 24.64 | Severe | 24.73 | 0.06 | 94307 | 99.99 | 99.42 | 88.35 | 3.51 | 3 | 99.40% | 78.80% | 0.00% | 0.58% | 4.96% | 2.56% | 0.17% | 0.04% | 0.01% |
| patients26 | Female | 2Years and 11Months | 2021/7/22 | 18.91 | Severe | 36.46 | 0.24 | 591683 | 100.00 | 100 | 100 | 99.95 | 3 | 99.60% | 80.20% | 0.00% | 0.39% | 3.95% | 1.82% | 0.05% | 0.04% | 0.01% |
| patients27 | Female | 9Years and 8Months | 2021/7/22 | 24.43 | Severe | 32.71 | 0.03 | 56271 | 99.95 | 97.9 | 54.35 | 0.07 | 3 | 95.90% | 70.40% | 0.00% | 4.12% | 13.20% | 9.34% | 0.96% | 0.07% | 0.02% |
| patients28 | Female | 2Years and 7Months | 2021/7/23 | 24.31 | Severe | 54.53 | 0.03 | 116974 | 99.35 | 98.95 | 95.47 | 12.2 | 14 | 99.20% | 75.50% | 0.00% | 0.83% | 8.40% | 6.01% | 0.36% | 0.05% | 0.01% |
| patients29 | Female | 2Years | 2021/7/29 | 25.12 | Severe | 58.43 | 0 | 540 | 1.50 | 0.16 | 0.11 | 0.05 | | 99.50% | 79.20% | 0.00% | 0.45% | 4.24% | 1.99% | 0.22% | 0.04% | 0.01% |
| patients30 | Male | 3Years and 1Months | 2021/7/30 | 26.56 | Severe | 58.18 | 0 | 1476 | 1.38 | 0.22 | 0.15 | 0.08 | | 97.50% | 69.00% | 0.00% | 2.46% | 13.60% | 10.80% | 0.64% | 0.06% | 0.01% |
| patients31 | Male | 7Years and 9Months | 2021/8/3 | 26.74 | Severe | 35.36 | 0.02 | 51345 | 99.24 | 94.57 | 44.73 | 0.01 | 14 | 99.90% | 81.50% | 0.00% | 0.12% | 2.57% | 0.18% | 0.01% | 0.05% | 0.01% |
| patients32 | Male | 3Years and 11Months | 2021/8/3 | 24.78 | Severe | 33.71 | 0.11 | 258412 | 100.00 | 100 | 99.97 | 96.47 | 3 | 99.80% | 80.90% | 0.00% | 0.16% | 2.88% | 0.49% | 0.01% | 0.05% | 0.01% |
| patients33 | Female | 4Years and 5Months | 2021/8/10 | 24.81 | Severe | 112.03 | 0 | 36109 | 98.34 | 78.22 | 17.65 | 0.01 | | 99.60% | 81.70% | 0.00% | 0.36% | 4.52% | 0.20% | 0.02% | 0.05% | 0.01% |
| patients34 | Female | 4Years and 8Months | 2021/8/14 | 26.58 | Severe | 31.89 | 0 | 3806 | 42.06 | 1.71 | 0 | 0 | | 99.90% | 82.10% | 0.00% | 0.15% | 2.41% | 0.16% | 0.02% | 0.05% | 0.01% |
| patients35 | Male | 1Months and 11Days | 2021/8/14 | 26.27 | Severe | 32.28 | 0 | 181 | 2.42 | 0.01 | 0 | 0 | | 99.80% | 80.80% | 0.00% | 0.17% | 2.66% | 0.17% | 0.02% | 0.05% | 0.01% |
| patients36 | Male | 5Years and 3Months | 2021/8/25 | 22.77 | Severe | 59.38 | 0 | 86571 | 99.32 | 98.72 | 86.69 | 1.61 | 14 | 99.90% | 81.40% | 0.00% | 0.13% | 2.44% | 0.18% | 0.01% | 0.05% | 0.01% |
| patients37 | Male | 12Years and 5Months | 2021/8/26 | 22.11 | Severe | 30.01 | 0.03 | 66669 | 99.99 | 98.9 | 69.22 | 0.03 | 3 | 99.80% | 80.20% | 0.00% | 0.20% | 2.69% | 0.27% | 0.02% | 0.06% | 0.01% |
| patients38 | Male | 1Years and 2Months | 2021/8/27 | 25.42 | Severe | 11.76 | 0 | 224 | 2.98 | 0 | 0 | 0 | | 99.90% | 80.90% | 0.00% | 0.11% | 2.45% | 0.18% | 0.01% | 0.05% | 0.01% |
| patients39 | Male | 10Years and 7Months | 2021/8/30 | 23.43 | Severe | 34.67 | 0 | 7801 | 64.66 | 8.75 | 0.01 | 0 | | 99.80% | 80.60% | 0.00% | 0.16% | 2.63% | 0.16% | 0.02% | 0.05% | 0.01% |
| patients40 | Female | 9Months and 28Days | 2021/4/15 | 26.78 | Severe | 33.61 | 0.03 | 78745 | 99.98 | 99.42 | 82.36 | 0.24 | 3 | 99.80% | 82.20% | 0.00% | 0.18% | 2.57% | 0.22% | 0.01% | 0.05% | 0.01% |
| patients41 | Female | 2Years and 3Months | 2021/5/7 | 38.32 | Severe | 8.08 | 0.01 | 6284 | 58.59 | 2.6 | 0 | 0 | | 99.70% | 74.40% | 0.00% | 0.30% | 2.31% | 0.07% | 0.01% | 0.00% | 0.00% |
| patients42 | Female | 7Years and 11Months | 2021/7/6 | 30.92 | Severe | 11.45 | 0.01 | 8152 | 62.70 | 5.21 | 0 | 0 | | 99.80% | 76.30% | 0.00% | 0.23% | 2.10% | 0.02% | 0.01% | 0.00% | 0.00% |
| patients43 | Male | 2Months and 6Days | 2021/4/19 | 30.09 | Severe | 11.25 | 0.02 | 15131 | 80.07 | 15.87 | 0.1 | 0 | 3 | 99.70% | 75.70% | 0.00% | 0.25% | 2.21% | 0.03% | 0.01% | 0.00% | 0.00% |
| patients44 | Female | 11Years and 6Months | 2021/5/24 | 25.73 | Severe | 13.06 | 0.08 | 65726 | 99.30 | 88.83 | 29.79 | 0 | 3 | 99.80% | 77.20% | 0.00% | 0.25% | 2.22% | 0.11% | 0.01% | 0.00% | 0.00% |
| patients45 | Female | 9Years and 8Months | 2021/6/28 | 27.13 | Severe | 14.84 | 0.12 | 114532 | 99.96 | 99.26 | 82.23 | 0.56 | 3 | 99.80% | 75.70% | 0.00% | 0.25% | 3.44% | 0.15% | 0.01% | 0.00% | 0.00% |
| patients46 | Female | 10Years and 10Months | 2021/5/4 | 15.57 | Severe | 11.63 | 11.43 | 8860053 | 100.00 | 100 | 100 | 99.77 | 3 | 99.60% | 65.10% | 0.00% | 0.42% | 15.50% | 13.00% | 0.02% | 0.00% | 0.00% |
