# Supplementary material for: Molecular epidemiology of Mycoplasma pneumoniae pneumonia in children, Wuhan, 2020–2022
Source: BMC Microbiol. 2024 Jan 17;24:23. doi: 10.1186/s12866-024-03180-0 (PMC10792977; doi:10.1186/s12866-024-03180-0)
Supplement: Supplementary file 5 — Additional file 5. [file 12866_2024_3180_MOESM5_ESM.pdf]

Table S3. Resistance genes detected in each sample.

Sampleid	Disease Severity	Sample Type	Tetracycline	Macrolide	Amikacin	Gentamicin	Kanamycin	Tobramycin	Lincosamides	Streptogramin	Cephalosporin	Beta-lactam	Chloramphenicol	Sulfonamide	Streptothricins	Florfenicol	Aminoglycoside	Erythromycin
0415-1	S	BALF																
0419-1	S	BALF					+											
0504-1	S	BALF		+			+											+
0507-1	S	BALF					+				+							
0524-1	S	BALF					+											
0615-1	S	BALF																
0624-2	S	BALF																
0628-3	S	BALF					+											
0706-1	S	BALF					+											
0803-1	S	BALF																
0803-2	S	BALF																
0810-1	S	BALF		+					+	+		+						
0814-1	S	BALF																
0814-2	S	BALF																
0825-2	S	BALF																
0826-1	S	BALF		+														
0827-2	S	BALF																
0830-1	S	BALF																
0202-1	M	OP	+	+	+	+	+	+	+	+	+	+						
0202-3	M	OP	+	+	+	+	+	+	+	+		+						
0204-5	M	OP	+	+	+	+	+	+	+	+	+	+						
0204-7	M	OP	+	+	+	+	+	+	+	+		+	+					
0301-2	S	OP	+	+	+	+	+	+	+	+		+	+					
0302-3	M	OP	+	+					+	+		+						
0323-5	M	OP	+	+	+	+	+	+	+	+		+	+	+	+			
0508-7	M	OP		+					+	+		+						
0524-3	M	OP		+					+	+		+		+				
0628-1	M	OP	+	+					+			+						
0628-2	S	OP	+	+	+	+	+	+	+	+	+	+						
0705-7	S	OP	+	+	+	+	+	+	+	+	+	+	+		+			
0705-11	M	OP		+		+			+			+						
0713-2	S	OP	+	+	+	+	+	+	+	+	+	+	+					
0713-6	M	OP	+	+	+	+	+	+	+	+		+						
0714-1	S	OP		+	+	+	+	+	+		+	+						
0714-3	M	OP	+	+	+	+	+	+	+	+	+	+						
0722-4	S	OP	+	+	+	+	+	+	+	+	+	+			+			
0722-10	S	OP	+	+		+	+		+	+	+	+		+				
0723-7	S	OP	+	+	+	+	+	+	+	+	+	+	+		+	+		
0724-5	M	OP	+	+								+						
0725-4	M	OP	+	+	+	+	+	+	+	+	+	+						
0728-3	M	OP	+	+	+	+	+	+	+	+	+	+	+					
0729-1	S	OP	+	+	+	+	+	+	+			+					+	
0730-2	M	OP	+	+	+	+	+	+	+	+		+						
0730-3	S	OP	+	+	+	+	+	+	+	+	+	+	+	+			+	
0804-1	M	OP	+	+					+	+								
0804-2	M	OP	+	+	+	+	+	+	+	+	+	+						
