# Supplementary material for: Molecular epidemiology of Mycoplasma pneumoniae pneumonia in children, Wuhan, 2020–2022
Source: BMC Microbiol. 2024 Jan 17;24:23. doi: 10.1186/s12866-024-03180-0 (PMC10792977; doi:10.1186/s12866-024-03180-0)
Supplement: Supplementary file 6 — Additional file 6. [file 12866_2024_3180_MOESM6_ESM.pdf]

Table S4. Species corresponding to the detected resistance genes.

Accession number	Antibiotics names	Organism
NG_047404.1	AMIKACIN;GENTAMICIN;KANAMYCIN;TOBRAMYCIN	Enterococcus faecium
NG_047408.1	AMIKACIN;GENTAMICIN;KANAMYCIN;TOBRAMYCIN	Campylobacter jejuni
NG_047418.1	AMIKACIN;KANAMYCIN	Enterococcus faecalis
NG_052450.1	AMINOGLYCOSIDE	Serratia marcescens BIDMC 50
NG_047633.1	BETA-LACTAM	Phocaeicola vulgatus
NG_047641.1	BETA-LACTAM	Capnocytophaga ochracea
NG_050145.1	BETA-LACTAM	Escherichia coli
NG_050162.1	BETA-LACTAM	Staphylococcus aureus
NG_050214.1	BETA-LACTAM	Escherichia coli
NG_059898.1	BETA-LACTAM	mixed culture bacterium PE_gF3SD01_32
NG_048596.1	CEPHALOSPORIN	Acidaminococcus fermentans
NG_048896.1	CEPHALOSPORIN	Capnocytophaga sputigena
NG_049393.1	CEPHALOSPORIN	Pseudomonas aeruginosa
NG_047562.1	CHLORAMPHENICOL	Limosilactobacillus reuteri
NG_047591.1	CHLORAMPHENICOL	Streptococcus pneumoniae
NG_047615.1	CHLORAMPHENICOL	Morganella morganii
NG_047655.1	CHLORAMPHENICOL	Pseudomonas aeruginosa
NG_047875.1	CHLORAMPHENICOL;FLORFENICOL	Salmonella enterica subsp. enterica serovar
NG_047246.1	GENTAMICIN	Plasmid R
NG_047251.1	GENTAMICIN	Citrobacter freundii
NG_047387.1	GENTAMICIN;KANAMYCIN;TOBRAMYCIN	Shigella sonnei
NG_059324.1	KANAMYCIN	Pseudomonas aeruginosa
NG_047920.1	LINCOSAMIDE	Bacteroides fragilis
NG_047924.1	LINCOSAMIDE	Streptococcus agalactiae
NG_047934.1	LINCOSAMIDE;STREPTOGRAMIN	Streptococcus agalactiae
NG_047797.1	MACROLIDE	Streptococcus pyogenes
NG_047798.1	MACROLIDE	Streptococcus agalactiae
NG_047801.1	MACROLIDE	Enterococcus faecium
NG_047804.1	MACROLIDE	Enterococcus faecium
NG_047825.1	MACROLIDE	Bacteroides fragilis
NG_047842.1	MACROLIDE	Limosilactobacillus reuteri
NG_047851.1	MACROLIDE	Corynebacterium jeikeium
NG_047853.1	MACROLIDE	Corynebacterium urealyticum DSM 7109
NG_047958.1	MACROLIDE	Streptococcus pneumoniae
NG_047960.1	MACROLIDE	Streptococcus sp. 'group G'
NG_047965.1	MACROLIDE	Streptococcus dysgalactiae subsp. equisimilis
NG_047966.1	MACROLIDE	Gemella haemolysans
NG_047980.1	MACROLIDE	Bacteroides fragilis
NG_048006.1	MACROLIDE	Streptococcus pneumoniae
NG_047324.1	STREPTOMYCIN	Escherichia coli
NG_047325.1	STREPTOMYCIN	Escherichia coli
NG_047393.1	STREPTOMYCIN	Enterococcus faecium
NG_047464.1	STREPTOMYCIN	Escherichia coli
NG_047466.1	STREPTOMYCIN	Vibrio cholerae
NG_052266.1	STREPTOMYCIN	Pseudomonas aeruginosa
NG_056002.2	STREPTOMYCIN	Salmonella enterica subsp. enterica serovar
NG_048072.1	STREPTOTHRICIN	Enterococcus faecium
NG_048082.1	SULFONAMIDE	Pseudomonas aeruginosa
NG_051852.1	SULFONAMIDE	Vibrio cholerae MO10
NG_048125.1	TETRACYCLINE	Streptococcus salivarius
NG_048126.1	TETRACYCLINE	Streptococcus parasanguinis
NG_048161.1	TETRACYCLINE	Shigella flexneri 2b
NG_048203.1	TETRACYCLINE	Streptococcus agalactiae
NG_048213.1	TETRACYCLINE	Enterococcus faecalis
NG_048217.1	TETRACYCLINE	Streptococcus pneumoniae
NG_048220.1	TETRACYCLINE	Clostridium perfringens
NG_048223.1	TETRACYCLINE	Bacillus sp. R89
NG_048230.1	TETRACYCLINE	Streptococcus cristatus
NG_048243.1	TETRACYCLINE	Clostridioides difficile 630
NG_048246.1	TETRACYCLINE	Streptococcus pneumoniae
NG_048247.1	TETRACYCLINE	Streptococcus suis
NG_048250.1	TETRACYCLINE	Streptococcus parauberis
NG_048252.1	TETRACYCLINE	Staphylococcus aureus subsp. aureus TW20
NG_048255.1	TETRACYCLINE	Streptococcus pneumoniae
NG_048264.1	TETRACYCLINE	Actinobacillus pleuropneumoniae
NG_048270.1	TETRACYCLINE	Prevotella ruminicola
NG_048271.1	TETRACYCLINE	Prevotella intermedia
NG_048281.1	TETRACYCLINE	Butyrivibrio fibrisolvens
NG_048286.1	TETRACYCLINE	Megasphaera elsdenii
NG_048291.1	TETRACYCLINE	Trueperella pyogenes
NG_048295.1	TETRACYCLINE	Rothia sp. T40-1
NG_048312.1	TETRACYCLINE	Streptococcus australis
NG_048318.1	TETRACYCLINE	Streptococcus australis
NG_051907.1	TETRACYCLINE	Pseudomonas aeruginosa
NG_056045.1	TETRACYCLINE	uncultured bacterium
NG_056046.1	TETRACYCLINE	uncultured bacterium
